# BEATRIX: An open source humanoid head platform for robotics teaching and research

**DOI:** 10.1016/j.ohx.2024.e00591

**Published:** 2024-10-09

**Authors:** Gorkem Anil Al, Nicholas Hedworth, Douglas Tilley, Samer Ahmed, Richmond Afeawo, Uriel Martinez-Hernandez

**Affiliations:** Multimodal Interaction and Robot Active Perception (inte-R-action) Lab, University of Bath, Bath, UK; Department of Electronic and Electrical Engineering, University of Bath, Bath, UK

**Keywords:** Robot head, Education, Open hardware, Humanoids, Human–robot interaction, Sensors

## Abstract

This paper introduces BEATRIX, a novel robotic head designed to bridge the gap between theoretical knowledge and practical experience in the field of robotics at universities. The BEATRIX robot comprises a head actuated by a neck-like mechanism with three stepper motors, two cameras and two microphones for acquisition of visual and audio information from the environment. The robot can be connected to any external computer for the design and implementation of algorithms for applications in human–robot interaction. The proposed robotic platform has been used successfully with undergraduate and master students implementing tasks such as face detection and tracking, sound detection and tracking, robot control and graphical user interfaces. This paper includes lists of all the robot components, assembly instructions, and links to all CAD and software files, facilitating replication and further exploration. The robot design and integration of visual and audio sensors enables the development of engaging educational tutorials and robot experiments, enhancing the teaching and learning experience in robotics.

## Graphical abstract

**Figure:**
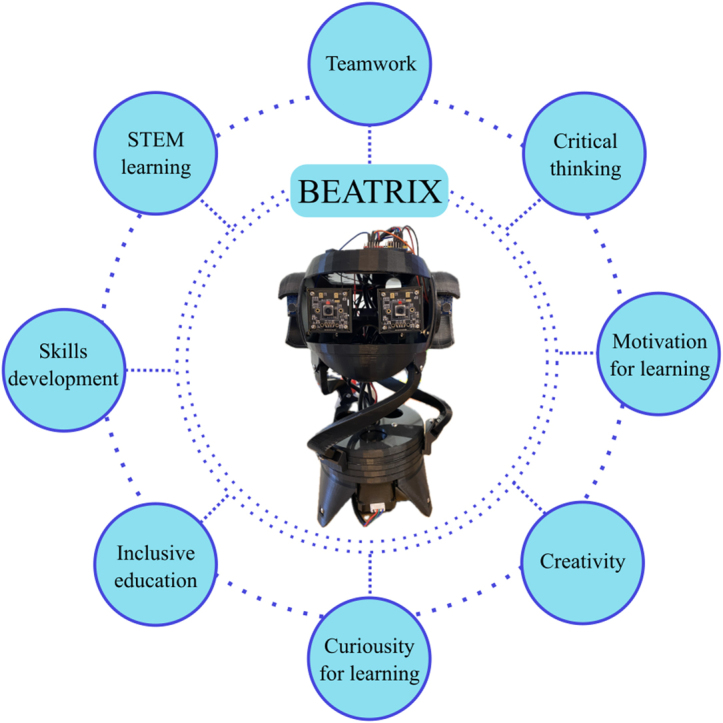

## ”Specifications table”

**Table:** 

Hardware name	BEATRIX humanoid robot

Subject area	• Educational tools and open source alternatives to existing infrastructure

Hardware type	• Robotics Engineering • Electrical engineering and computer science

Closest commercial analog	Closest commercial analag: EZ-InMoov Humanoid Head Differences: BEATRIX uses standard and affordable microcontrollers, sensors and motors and is open hardware and open source

Open source license	CERN-OHL-P-2.0 for the hardware Apache 2.0 for the software

Cost of hardware	£273.49

Source file repository	https://doi.org/10.17605/OSF.IO/T8EGC

OSHWA certification UID 0.1 cm	UK000065

## 1 Hardware in context

Robotics education at universities plays a crucial role in preparing students for the rapidly evolving technological landscape of the future in many fields of engineering [1], [2], [3]. The use of robotics in education gives students a platform to explore and gain a diverse set of skills ranging from mechanical design and electronics to programming, control and artificial intelligence. These skills are in high demand in industries such as manufacturing, healthcare, transportation, agriculture, and service robotics.

The majority of universities opt to utilize simulators as a means to demonstrate theoretical knowledge in robotics education [4], [5], [6]. This approach is used given that simulators offer several advantages, including cost-effectiveness and minimal maintenance requirements. By leveraging simulators, universities can provide students with an interactive learning experiences without the need for physical equipment. However, physical robots offer a hands-on, tangible and unique learning experience that simulators cannot replicate. Interacting with physical hardware allows students to gain a deeper and richer understanding of robotics concepts, such as mechanics, electronics, programming, control and sensor integration, by observing how their actions directly affect the robot’s behavior. Moreover, physical robots encounter real-world challenges and limitations that simulators may not accurately replicate, inspiring creativity and innovation as students investigate and explore solutions designing, building, and experimenting with their own robotic systems physically.

Various types of robots are utilized to teach different disciplines within robotics education. These include industrial robots such as Universal Robots [7], [8], [9] and Franka Emika Panda [10], as well as humanoid robots like NAO [11], EZ-InMoov Humanoid Head [12], OHBOT [13] and iCub humanoid robot [14], [15], and mobile robots, which can be found in small scale and low cost, particularly in teaching swarm robotics concepts [16], [17], [18]. Additionally, companies like Quanser [19] and Festo [20] offer educational and research-focused solutions in control systems, robotics, and mechatronics. However, while commercial robots offer advanced features and capabilities, they often come with significant drawbacks for educational settings. Firstly, the cost of these robots make them unaffordable for many educational institutions, making it challenging to provide access to each student or to purchase multiple units for group learning activities. Commercial robots typically come with predefined functionalities, closed platforms and limited opportunities for customization. This lack of flexibility can hinder the student ability to explore diverse robotics concepts and apply their creativity to solve unique challenges. Furthermore, the proprietary nature of commercial robots restrict access to underlying hardware and software components, limiting the student ability to delve deeper into the mechanics and algorithms that govern robotic systems.

In our paper, we introduce the BEATRIX robotic platform, a robot head with an open-source hardware and software solution designed for university educational and research purposes. There are not many robot heads developed specifically for university students. Therefore, BEATRIX aims to fill this gap by providing a customizable, accessible, and affordable platform for students to explore and experiment with various robotics concepts and applications. The BEATRIX robot offers a unique opportunity for students to engage, explore and implement a variety of theoretical and practical robotics engineering aspects, fostering a deeper understanding and encouraging curiosity, teamwork, experiment design and innovative solutions and problem-solving.

## 2 Hardware description

**Fig. 1:**
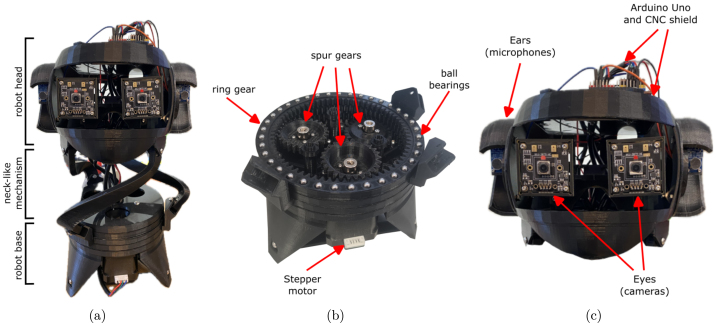
Overview of mechanical system of the BEATRIX robot. (a) BEATRIX robot with indication of all components. (b) Mechanism of the robot base to actuate the neck-like section. (c) Robot head composed of microphones and cameras.

The BEATRIX robot, shown in Fig. 1(a), is an open-source hardware and software platform for university education and research. This platform was designed with mechanical, electronics, sensors and computational aspects to teach robotics engineering with applications such as object and sound detection and tracking, perception and action for human–robot interaction and autonomous systems, where the robot perceives information from the environment, employing cameras and microphones to make decision and perform appropriate actions. Currently, there are only a few robot head platforms for robotics teaching and research. While other robots focus on developing programming skills based on the use of proprietary modules, they often come with pre-assembled parts and limited modularity, making it harder for students to delve into the hardware and software modifications at a deeper level. In contrast, our BEATRIX robot platform uses off-the-shelf components, assembled in a modular approach and full open hardware and software, that allows students to explore and develop multiple engineering skills. These engineering skills include understanding the assembly of mechanical components, electrical and electronic units to power and communicate multiple devices, kinematics, motor control and geared systems, data collection and processing at different levels, connection and communications with sensors, 3D printing process and overall system integration. Regarding research aspects, the BEATRIX robot offers a platform for researchers to explore, implement and validate easily and affordably computer vision methods, audio recognition methods, bioinspired mechanism such as the current neck-like approach used in our robot, intelligent motor controllers and multimodal sensing for a variety of applications. Furthermore, the modular approach of BEATRIX with off-the-shelf components, allows the user to replicate this robot, but also, upgrade it with different actuators and links for the neck, cameras, microphones, units with higher computational resources and embedded array of sensors for data collection and perception.

### 2.1 Mechanical system

The mechanical design of BEATRIX resembles that of a parallel robot. Its structure comprises a 3D printed neck-like mechanism supporting the robot’s head, while a fixed base, also 3D printed, houses the actuator mechanism. Within the fixed base, three NEMA-17 stepper motors (42 mm×40 mm) drive the system. Each motor is connected to a spur gear via a flange coupling, with the 3D printed spur gears meshing with corresponding ring gears (Fig. 1(b)). These ring gears are arranged sequentially, and their rotational motion is facilitated by ball bearings placed between them. The resultant motion is transmitted to the neck-like mechanism (mobile platform) through connection rods, affixed to both the fixed and mobile platforms using groove ball bearings.

**Fig. 2:**
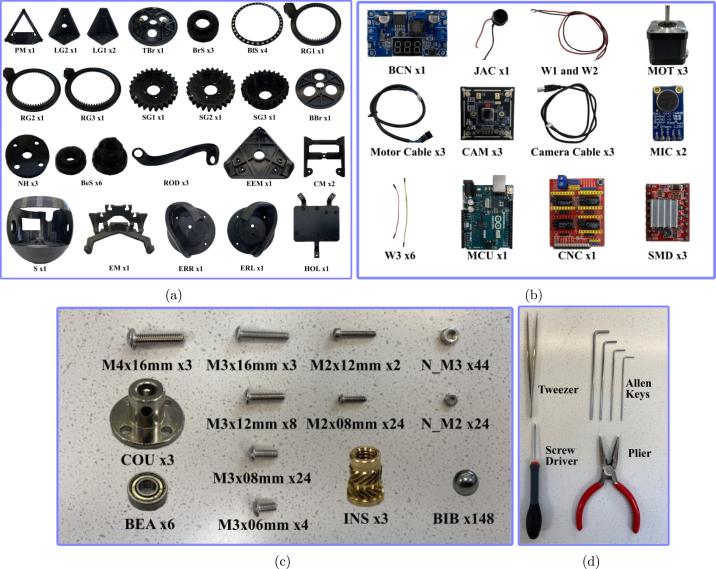
Overview of all components to assemble the robot. (a) 3D printed robot parts. (b–c) Electronic and mechanical components used in the robot. (d) Tools used for the assembly process.

The robot head is formed by 3D printed skull manufactured using polylactic acid (PLA) material with an FDM 3D printer. The skull provides a lightweight yet durable housing for BEATRIX’s internal components: (i) the eye mechanism, (ii) left and right ears, (ii) microcontroller, (iii) motor drivers and (iv) ADC for audio sensing. The robot head and its components are shown in Fig. 1(c). The entire robot assembly, comprising the fixed base and mobile platform, is supported by three rod parts, completing the mechanical assembly of BEATRIX. All 3D printed parts used in the assembly of this robot platform are shown in Fig. 2(a).

### 2.2 Electrical system

The electrical components of BEATRIX, shown in Fig. 2(b), can be categorized into two main groups: (i) power components responsible for providing and regulating the necessary electrical energy for operation, and (ii) sensor components for gathering data and facilitating interaction with the robot’s environment.

The NEMA-17 stepper motor operates at a voltage of 12 V. To supply power to the motor, a 12 V power supply is utilized, which is connected through a LM5296 DC–DC buck converter. While the motor can be directly powered without the use of a DC–DC buck converter, we have chosen to incorporate the converter into the system to allow future adjustments and enhancements to the functionality of the BEATRIX robot.

The output power from the DC–DC buck converter is distributed to the Computer Numerical Control (CNC) shield module, which is mounted on the Arduino Uno microcontroller board located atop BEATRIX. The CNC shield is Arduino-compatible, and it simplifies the process of controlling the stepper motors of the robot base by providing a standardized motor driver interface. This CNC shield has four slots for plugging in stepper motor driver modules, and three of them are used by three A4988 motor drivers utilized for precise and efficient control and drive of the current needed to actuate the NEMA-17 stepper motors of BEATRIX robot. The A4988 features adjustable current limiting, empowering users to fine-tune the motor current for optimal performance and prevent overheating. Additionally, the CNC shield offers the flexibility to select various motor step sizes, including full-step, half-step, quarter-step, and more, meeting specific application requirements of BEATRIX.

Our robotic platform is equipped with sensors for visual and auditory data collection from the surrounding environment. Visual sensing is facilitated by two Sony IMX179 CMOS cameras integrated into the eye mechanism. This 8 Megapixels USB Camera with microphone, boasting an 8000K pixels driver-free USB port, enhances its sensory capabilities with a resolution of up to 3264 × 2448 pixels. The cameras in the BEATRIX robot offer compatibility with various operating systems including Windows, iOS, Android, and Linux, ensuring seamless integration into different environments.

The BEATRIX auditory system is facilitated by two MAX9814 microphones embedded within 3D printed ear designs on both the left and right sides of the robot’s skull. Each microphone features analog outputs and is seamlessly integrated into the BEATRIX’s design, as illustrated in Fig. 1(c). Operating at the Arduino Uno power level of 5 V, these microphones offer straightforward usability and compatibility. Users can directly access the analog output for post-processing tasks using the Arduino UNO analog pins or opt to connect the microphone to an intermediate Analog-to-Digital Converter (ADC) to digitize the output, which can then be transmitted to the microcontroller through an I2C bus. Conveniently, the microphones can be easily connected to the CNC shield using the pins labeled ‘hold’ and ‘abort’, which correspond to analog pins A1 and A0 of the Arduino Uno microcontroller, respectively.

The Arduino Uno serves as the central processing unit, or ‘brain’, of BEATRIX. The Arduino Uno microcontroller is programmed with a set of motor control functions that can be called by any external program for precise control of robot actions via the motors located in the robot base. The BEATRIX humanoid robot offers students and researchers a variety of utilities such as the following:

• Replicate and manufacture BEATRIX using the comprehensive information provided in the paper, including CAD files and readily available commercial electronic and mechanical products.
• Open-source design of BEATRIX for easy customization and hardware modification to suit specific educational objectives. For instance, researchers can integrate additional sensors into the head design or upgrade the actuator mechanism as needed.
• BEATRIX offers versatility in programming options, allowing researchers to utilize various programming boards or single-board computers such as Raspberry Pi. Additionally, the robot can be controlled through programs developed in popular languages such as MATLAB, Python and C++, enabling a wide range of robotic applications and experimentation.

In this first version of the BEATRIX robot, the purpose was to process data using a PC, and thus, an advanced controller for driving the motors was not required. Therefore, we chose Arduino, which offers a readily available microcontroller with a variety of libraries ready for programming and working with different components. Thus, the BEATRIX’s design follows a modular approach, where the set of control commands (Arduino microcontroller) and motor drivers (CNC shield) are separated. Robot actions are generated in programs such as in C++ and Python implemented in an external computer connected to Arduino to generate the corresponding control commands. This modular approach allows a better understanding of the different levels of processing performed by the robot. Additionally, a modular design is useful for debugging, and easier and affordable replacement of components. In future work, we plan to upgrade the ‘brain’ of BEATRIX with a processing unit with higher computational capacity, e.g., Raspberry PI. This will allow the user to collect and process data and implement robot actions locally and using different programming languages, without the need of an external computer, but still keeping the modular approach of our robot.

## 3 Design files summary

All the design files used in the BEATRIX robot platform are listed below. The description of each file and its location in the Open Science Framework (OSF) repository are shown in Table 1.

1. **Bottom Bracket (BBr)** — Base layer of the rotary mechanism. Attaches to legs and motors.
2. **Bearing Spacer (BeS)** — Used to secure the connecting rods to the rotary mechanism and the head.
3. **Ball Separator (BlS)** — Keeps ball bearings evenly spaced within the rotary mechanism while still being able to roll freely.
4. **Bracket Spacer (BrS)** — Extends the height of the BBr pillars to prevent the TBR from compressing the mechanism too much when attached (allowing for smooth movement).
5. **Camera Mount (CM)** — To hold the cameras. One per camera.
6. **End Effector Mount (EEM)** — Attaches to the base of the skull. Used to connect to the connecting rods that are driven by the rotary mechanism.
7. **Eye Mount (EM)** — Placed within the SKL. Both camera mounts are secured to this.
8. **Left & Right Ear (ERL, ERR)** — Hold the microphones securely in place on the head.
9. **PCB Holder (HOL)** — Secured to the top of SKL. Holds the control electronics for getting sensor input and driving the motors.
10. **Base (LG1)** — Standard leg design, no modifications. Two of these are used at the base of the robot.
11. **Base Power Input (LG2)** — Leg design with a hole in it for mounting the DC Jack for power input. Only one is required for the build.
12. **Nut Holder (NH)** — Holds the nuts that are used to attach the spur gears to the motor shafts.
13. **Power Mount (PM)** — Holds the DC–DC converter at the base of the assembly.
14. **Ring Gear 1,2,3 (RG1, RG2, RG3)** — Outer gears of the rotary mechanism. Arranged from tallest at the bottom, to shortest at the top of the assembly.
15. **Connecting Rod (ROD)** — Joins the rotary mechanism to the bead.
16. **Spur Gear 1,2,3 (SG1, SG2, SG3)** — Internal gears of the rotary mechanism. Arranged specifically to align with each RG.
17. **Skull (SKL)** — Holds all sensors and control electronics.
18. **Top Bracket (TBr)** — Covers the rotary mechanism, securing the free-moving parts.

**Table 1:** Hardware design files.

Designator	Design filename	File type	Open source license	Location of the file
BBr	Bottom-Bracket	CAD file	CERN-OHL-P-2.0	https://osf.io/pqvxf
BeS	Bearing Spacer	CAD file	CERN-OHL-P-2.0	https://osf.io/msk4a
BlS	Ball Separator	CAD file	CERN-OHL-P-2.0	https://osf.io/9jsfx
BrS	Bracket Spacer	CAD file	CERN-OHL-P-2.0	https://osf.io/2rkym
CM	Camera Mount	CAD file	CERN-OHL-P-2.0	https://osf.io/h2wd5
EEM	End Effector Mount	CAD file	CERN-OHL-P-2.0	https://osf.io/95qzh
EM	Eye Mount	CAD file	CERN-OHL-P-2.0	https://osf.io/4xehs
ERL	Left Ear	CAD file	CERN-OHL-P-2.0	https://osf.io/xzah7
ERR	Right Ear	CAD file	CERN-OHL-P-2.0	https://osf.io/ugrh4
HOL	PCB Holder	CAD file	CERN-OHL-P-2.0	https://osf.io/sf2q9
LG1	Base	CAD file	CERN-OHL-P-2.0	https://osf.io/sd9v7
LG2	Base Power Input	CAD file	CERN-OHL-P-2.0	https://osf.io/y9kcs
NH	Nut Holder	CAD file	CERN-OHL-P-2.0	https://osf.io/csy97
PM	Power Mount	CAD file	CERN-OHL-P-2.0	https://osf.io/4h2a5
RG1	Ring Gear 1	CAD file	CERN-OHL-P-2.0	https://osf.io/ubh6w
RG2	Ring Gear 2	CAD file	CERN-OHL-P-2.0	https://osf.io/zjxgc
RG3	Ring Gear 3	CAD file	CERN-OHL-P-2.0	https://osf.io/se23f
ROD	Connecting Rod	CAD file	CERN-OHL-P-2.0	https://osf.io/xr4bh
SG1	Spur Gear 1	CAD file	CERN-OHL-P-2.0	https://osf.io/xs9qw
SG2	Spur Gear 2	CAD file	CERN-OHL-P-2.0	https://osf.io/asztb
SG3	Spur Gear 3	CAD file	CERN-OHL-P-2.0	https://osf.io/exkv2
SKL	Skull	CAD file	CERN-OHL-P-2.0	https://osf.io/gzehu
TBr	Top Bracket	CAD file	CERN-OHL-P-2.0	https://osf.io/dvn58

## 4 Bill of materials

The bills of materials are given in Table 2, Table 3 for mechanical components and electronic components, respectively. All the components shown in Table 1 can be manufactured using PLA material with a standard 3D printer. Using an infill of 20% for all the parts, 754 g of PLA was required for 3D printing, equating to £23.26. The cost of materials and printing given in this paper do not include value-added tax (VAT) and is based on the version of the BEATRIX robot presented in this paper. However, the authors note that some of the costs can be reduced by using a lower infill percentage for 3D printing and sourcing materials and parts from alternative vendors.

### 4.1 BOM of mechanical components

The description of each mechanical component used in this robot is shown in Table 2.

**Table 2:** BOM of mechanical components.

Designator	Component description	Number	Cost per unit	Total cost	Source of materials	Material type
BEA	696ZZ Budget Metal Shielded Deep Groove Ball Bearing 6 × 15 × 5 mm	6	£2.39	£14.34	Simply Bearings	Steel
BlB	4.5 mm Diameter Stainless Steel Ball Bearings	148	£0.09	£13.31	Simply Bearings	Stainless Steel
COU	Motor Flange Coupling (5mm Inner Diameter, H12 × D10)	3	£3.25	£9.74	Amazon	Iron Plating
INS	M4 Brass Threaded Insert, 7.1 mm diameter × 5.6 mm depth, 8.2 mm length	3	£0.34	£1.01	RS Components	Brass
M2xZ8	M2 × 8 mm Socket Button Head Screws	12	(Pack of 100) £24.39	£5.85	GWR-Fasteners	Stainless Steel
M2xZ2	M2 × 12 mm Socket Cap Head Screws	14	(Pack of 100) £5.00	£0.10	GWR-Fasteners	Stainless Steel
M3xZ6	M3 × 6 mm Socket Button Head Screws	12	(Pack of 100) £2.22	£0.27	GWR-Fasteners	Stainless Steel
M3xZ8	M3 × 8 mm Socket Button Head Screws	30	(Pack of 100) £2.37	£0.71	GWR-Fasteners	Stainless Steel
M3xZ2	M3 × 12 mm Socket Button Head Screws	8	(Pack of 100) £2.82	£0.23	GWR-Fasteners	Stainless Steel
M3xZ6	M3 × 16 mm Socket Button Head Screws	6	(Pack of 100) £3.09	£0.19	GWR-Fasteners	Stainless Steel
M4xZ6	M4 × 16 mm Socket Button Head Screws	3	(Pack of 100) £4.11	£0.12	GWR-Fasteners	Stainless Steel
N_M2	M2 Full Nuts	26	(Pack of 100) £3.00	£0.78	GWR-Fasteners	Stainless Steel
N_M3	M3 Full Nuts	44	(Pack of 100) £2.00	£0.88	GWR-Fasteners	Stainless Steel

### 4.2 BOM of electrical components

The description of each electrical component used in this robot is shown in Table 3.

**Table 3:** BOM electrical components.

Designator	Component description	Number	Cost per unit (£)	Total cost (£)	Supplier	Supplier part no.
BCN	DFR0379 DC–DC Buck Converter	1	£3.94	£3.94	Farnell	3769933
CAM	USB Camera Sony IMX179 CMOS	2	£31.23	£62.46	Farnell	3769901
CNC	Arduino CNC Shield	1	£7.71	£7.71	123-3D	DRW00016
JAC	2.1 × 5 mm Panel Mount DC Jack	1	£0.24	£0.24	Switch Electronics	210 009
JUM	Jumper (Busbar)	6	£1.37	£8.22	Farnell	2579721
MCU	Arduino Uno	1	£14.65	£14.65	Farnell	2285200
MIC	MAX9814 Microphone	2	£6.27	£12.54	DigiKey	1528-1713-ND
MOT	Stepper Motor, 1.8 Deg, 5MM Bipolar	3	£11.45	£34.35	Farnell	3879694
PLG	UK Plug to IEC C13 Socket	1	£2.54	£2.54	Farnell	4159175
PSU	Power Supply, 12 V, 2.5 A	1	£27.59	£27.59	Farnell	2630905
SHR	3.2 mm to 1.6 mm Heat Shrink	1	(100 m) £16.73	£0.02	Farnell	1008430
SMD	A4988 Stepper Motor Driver	3	£8.99	£26.97	Amazon	B0793K9KF8
W1	20 AWG Wire — Red	500 mm	(30.5 m) £10.00	£0.16	Farnell	2290836
W2	20 AWG Wire — Black	500 mm	(30.5 m) £12.72	£0.21	Farnell	2290845
W3	20 mm Jumper Wires (Female-to-female)	6	(20 wires) £3.72	£1.12	Farnell	3617779

## 5 Build instructions

The BEATRIX humanoid robot has been designed and developed in the Multimodal inte-R-action lab and used for teaching, lab activities and final projects in undergraduate and master units at the University of Bath. This robot has been designed to be modular to allow the reuse of parts as well as simplifying modifications for future designs and versions of the robot platform. Fig. 2 shows all the 3D printed parts, mechanical and electronic components and tools required to build the BEATRIX robot.

BEATRIX’s modular design consists of many components of varying sizes and quantities. Thus, performing the assembly process with precision is crucial to ensure optimal performance and operational reliability. Section 5.1 highlights several critical and technically challenging aspects of the assembly procedure, where meticulous attention is necessary to avoid potential issues affecting the robot’s functionality.

### 5.1 Build considerations

Some areas of the build process have been identified as potentially challenging. This section highlights these difficulties, how they may arise, and authors’ suggestions for overcoming them.

Rotary Mechanism: When placing the ball bearings into the ball separators, manual placement can be challenging, leading to ball bearings falling into the assembly or even displacing the entire assembly, dislodging the arms, and causing most ball bearings to fall out of place. With the current design, the risk of misplacing the ball bearings during installation remains inherent. To mitigate this risk, it is advisable to use a specialized tool, such as long-nose pliers, to handle and place the ball bearings, thereby minimizing the likelihood of accidental contact with the assembly. Additionally, positioning the assembly within a large tray can help capture any fallen parts should the assembly be disturbed. In future work, this risk could be further minimized by integrating the ball separators with the channels they sit on, improving stability and reducing the chances of displacement.

It is also important to make sure there is smooth contact and correct coupling between all the 3D printed parts — this is particularly noticeable in the rotary mechanism but applies to the entire assembly. For each part that has been 3D printed, remove all the support material, clear out the holes that have any stringing, and file down all the rough edges. The spur gears and ring gears need their teeth and grooves to be clear to allow for smooth engagement with the rest of the rotary mechanism.

Spur Gear Assembly: The nut holders and flange couplers must first be mounted on the motor shaft when installing the spur gears. Only after this step can the corresponding spur gear be positioned on the motor and all components screwed together. An issue that can arise at this point is keeping the parts together when aligning the holes to tighten the screws. Due to limited space, it is helpful to us a thin tool to leverage the nut holder up to the coupler and spur gear.

There is scope to later modify the design of the spur gears to have an access hole, which can be used to tighten the coupler to the motor. Meaning that the nut holder, coupler and spur gears can be assembled and then placed on the motor afterwards.

Stepper Driver Configuration: Proper configuration of the stepper motors is crucial because the gear mechanism limits the robot’s rotational speed, which can result in jerky movements. Additionally, slow motion is undesirable in the applications proposed in the paper. To balance smoothness and speed, we recommend connecting the jumpers to the first and second pin pairs, leaving the third pair unconnected. This setup enables 1/8 stepping mode, providing smooth yet swift head motion for the robot.

### 5.2 Wiring

*Required parts: JAC, SHR, W1, W2*

**Fig. 3:**
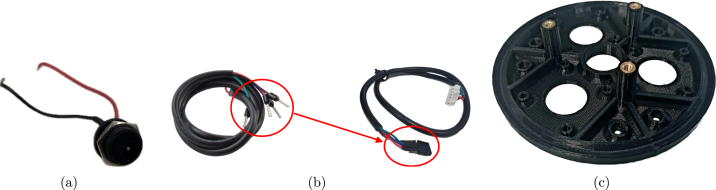
Description of wiring and bracket. (a) DC jack with wiring. (b) Motor cable with modifications. (c) Bottom bracket with head-set inserts.

1. Cut 70 mm lengths of W1 and W2 and solder them to the DC jack on their respective terminals — in this case the tip is positive and the sleeve is negative. Use heat shrink to protect the connections.
2. Remove ferrules from the motor cables and connect the bare ends to a 1 × 4 socket header (2.54 mm spacing) in the order: RED, BLUE, GREEN, BLACK. Again, be sure to apply heat shrink to the connections. The wiring process is shown in Fig. 3.

### 5.3 Bottom bracket

*Required parts: BBr, INS x3*

1. For each pillar of the BBr, place a heat-set insert on the pillar and use a soldering iron to gently push it in, until it sits flush with the pillar. See Fig. 3.

### 5.4 Head assembly

*Required parts: EEM, ERL, ERR, SKL, MIC (x2), W3 (x6), M2x08 (x4), M3x06 (x8), M3x08 (x6), N_M2 (x4), N_M3 (x14)*

**Fig. 4:**
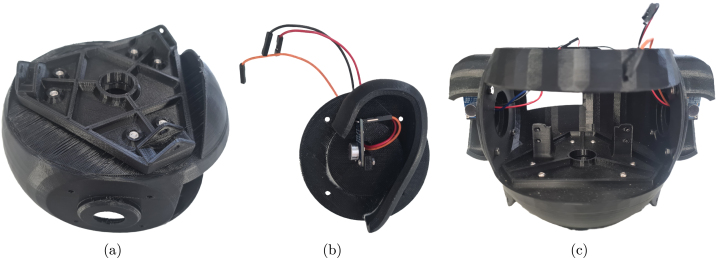
Primary stages of head assembly (a) Underside of skull with end effector mount attached (b) Left ear assembly with microphone and wires (c) Front view of skull with ears attached.

1. Attach the skull to the end effector mount using 6x M3x08 screws and M3 nuts.
2. For each ear, attach a microphone to the ear with 2x M2x08 screws and nuts.
3. Mount the ears on the head using 4x M3x06 screws and nuts each.
4. Connect a jumper wire to VDD, GND, and OUT on each microphone and pass them through the hole of the ear. The head assembly process is shown in Fig. 4.

### 5.5 Power mount assembly

*Required parts: DC Jack with wires, PM, BCN, W1 (400 mm), W2 (400 mm), M3x06 (x4), N_M3 (x4)*

**Fig. 5:**
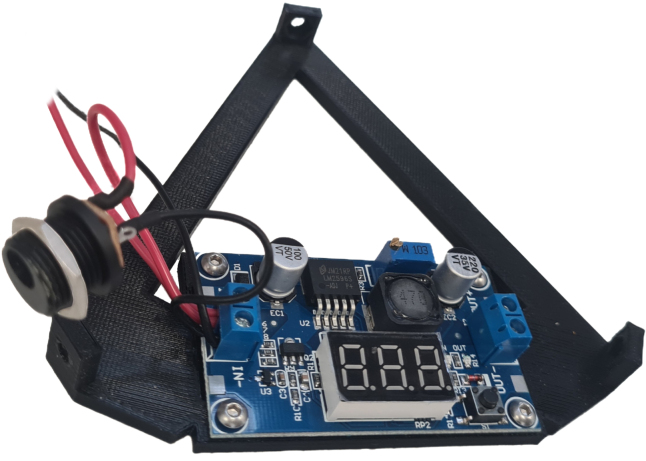
Power Mount with voltage converter attached, and power wiring connected.

1. Screw DC–DC converter onto power mount, with the input on the left and output on the right.
2. Connect the bare ends from the DC jack to the DC–DC converter along with 400 mm lengths of W1 and W2 in the matching terminals. See Fig. 5.

### 5.6 Base assembly

*Required parts: Power Mount Assembly, BBr, BrS (x3), LG1 (x2), LG2 (x1), MOT (x3), M3x08 (x21), N_M3 (x9)*

**Fig. 6:**
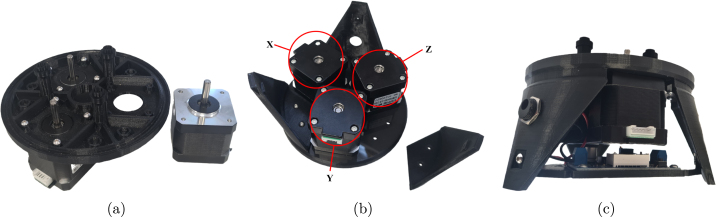
Base assembly (a) Bottom bracket with motors being attached (b) legs added to the assembly; motors labeled “X”, “Y”, “Z” clockwise from LG2 (c) Power Mount added to the assembly.

1. Place a bracket spacer (BrS) on each pillar of the bottom bracket (BBr), covering the heat-set inserts.
2. Using 12x M3x08 screws, attach the motors to the underside of the bottom bracket with their connectors pointing outwards.
3. Attach LG1 (x2) and LG2 to the bottom bracket with M3x08 screws and M3 nuts.
4. Turn the assembly upside down and attach the power mount assembly to the bottom bracket with an M3x08 screw for each leg, placing LG2 closest to the input of the DC converter.
5. Pass DC jack through hole of LG2 and secure it in place with a nut. The base assembly process is shown in Fig. 6.

### 5.7 Gear mechanism assembly

*Required parts: Base Assembly, BlS (x4), NH (x3), RG1, RG2, RG3, SG1, SG2, SG3, TBr, BlB (x148), COU (x3), M2x12 (x12), M4x16 (x3), Motor Grub Screws, N_M2 (x12)*

1. For each NH, place the four required M2 nuts.
2. Place an NH on each motor with the nuts facing downwards.
3. Using the grub screws that are provided with the shaft couplers, secure the couplers to the motors so that they are approx. 2 mm above the top of the motor shaft.

**Fig. 7:**
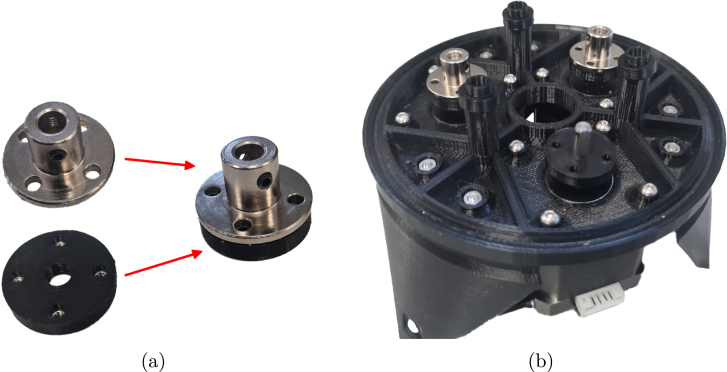
Rotary mechanism initial stage (a) Nut holder and motor coupler alignment (b) base assembly with nut holders and coupler being placed.

4. Place BlS on the base and then RG1 on BlS.
5. Place SG1 (concave side down) on one of the motors and check that it aligns with the teeth of RG1 (preferably slightly lower). If it does not align well, re-adjust the motor shaft coupler until the alignment is as desired.
6. Remove RG1 and BlS.
7. Push the nut holder up to the coupler, and line it up with the gear and coupler holes. Then screw all the pieces together with M2x12 screws.

**Fig. 8:**
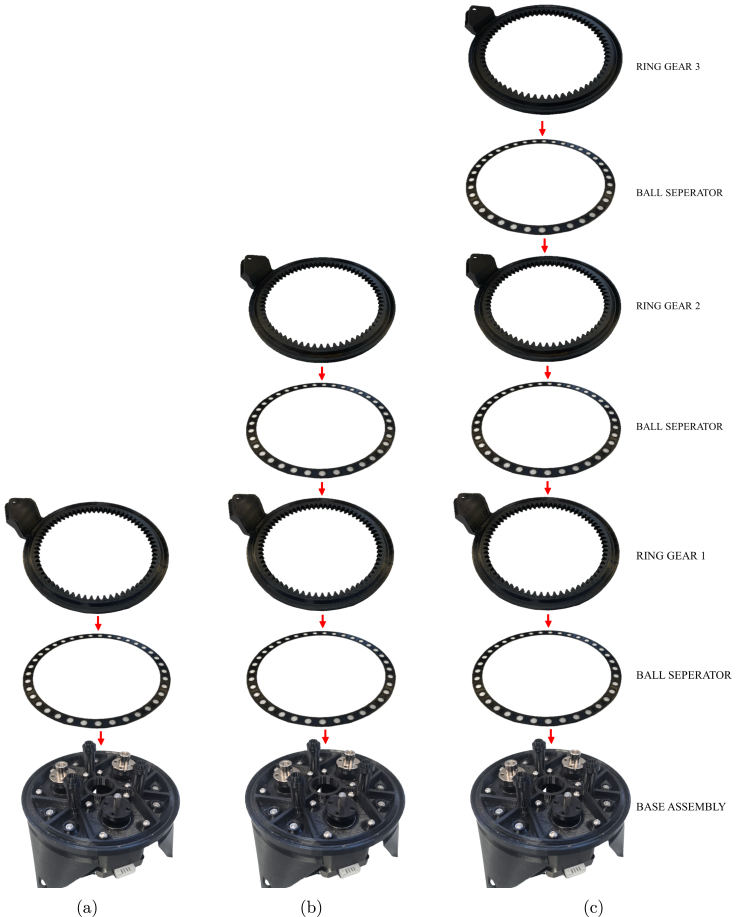
Stages of rotary mechanism assembly for spur gear alignment (a) First layer using Ring Gear 1 (b) Second layer with Ring Gear 2 placed on the first layer (c) All layers, Ring Gear 3 on top .

**Fig. 9:**
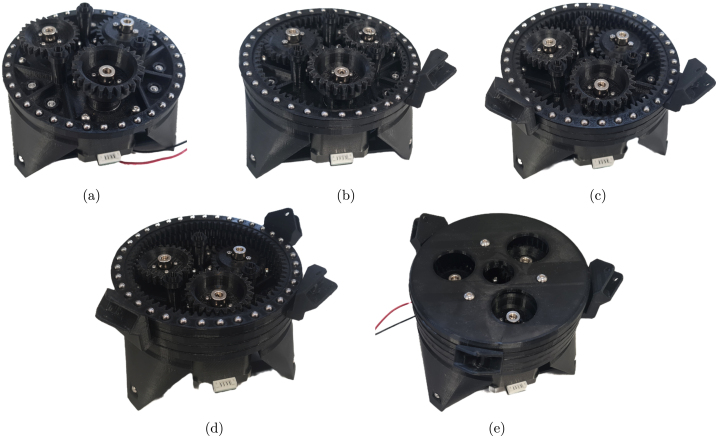
Final rotary mechanism assembly stages with ball bearings (a) First layer — ball bearings placed in base assembly (b) Second layer — ball bearings placed on ring gear 1 (c) Third layer — ball bearings placed on ring gear 2 (d) Fourth layer — ball bearings placed on ring gear 3 (e) Mechanism covered with top bracket.

8. Place BlS, then RG1, then BlS, then RG2 on the Base Assembly.
9. Place SG2 (concave side up) on the next motor going counter-clockwise and check that it aligns with the teeth of RG2. If it does not align well, re-adjust the motor shaft coupler until the alignment it as desired (the arms and spacers can be removed to allow access to the grub screw).
10. Remove all spacers and Ring Gears. Push the nut holder up, and line it up with the gear and coupler holes. Then screw all the pieces together with m2x12 screws.
11. Place BlS, then RG1, then BlS, then RG2, then BlS, and RG3 on the Base Assembly.
12. Place SG3 (concave side up) on the last motor and check that it aligns with the teeth of RG1. If it does not align well, re-adjust the motor shaft coupler until the alignment it as desired (the arms and spacers can be removed to allow access to the grub screw)
13. Remove all spacers and Ring Gears. Push the nut holder up, and line it up with the gear and coupler holes. Then screw all the pieces together with m2x12 screws.
14. Place a ball separator on the base, followed by ball bearings in all the holes, and RG1.
15. Place a ball separator on RG1, followed by ball bearings in all the holes and then RG2.
16. Place a ball separator on RG2, followed by ball bearings in all the holes and then RG3.
17. Place a ball separator on RG3, followed by ball bearings in all the holes and then TBr.
18. Fasten the top bracket onto the assembly with M4x16 screws. The step-by-step assembly of the gear mechanism and final rotary mechanism are illustrated in Figs. 7, 8 and 9.

### 5.8 Head attachment

*Required parts: Gear Mechanism Assembly, Head Assembly, BeS (x6), ROD (x3), BEA (x6), M3x16 (x6), N_M3 (x6)*

**Fig. 10:**
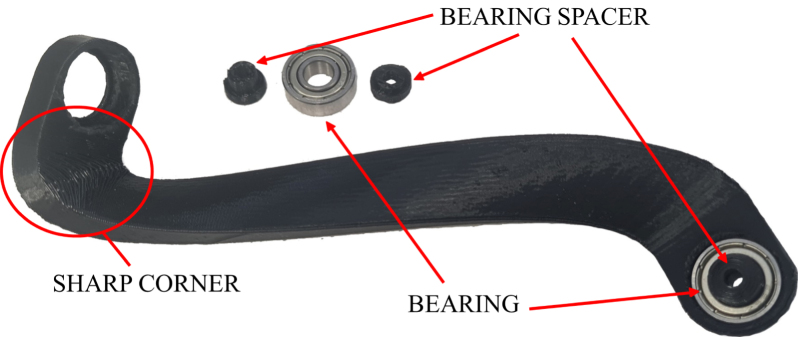
Connecting rod and accompanying components — bearings and bearing spacers.

**Fig. 11:**
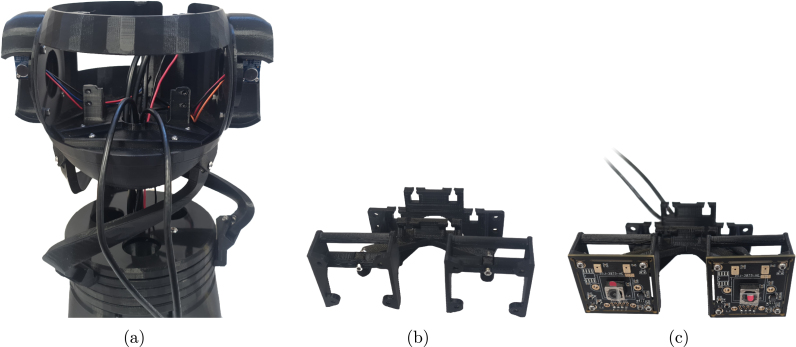
Camera assembly (a) Shows the BEATRIX head attached to the neck, with all wires passed through from below (b) Camera mounts attached to the eye holder (c) Cameras assembles with the camera mounts and eye holder.

1. Place a bearing into each hole of the 3 connecting rods.
2. Place the ends of the bearing spacer on each side of a bearing.
3. With bearing spacers in place, put the end of each rod with the sharp corner into each RG attachment. Secure them with M3x16 screws and nuts.
4. Repeat the previous steps for the other ends of the connecting rods and attach them to the head assembly.
5. Attach cables to motors and pull through the holes in the center of the assembly and out of the head.
6. Pass the JLC connections of the camera cables through the assembly.
7. Pull 400 mm wires through the center hole. See Fig. 10, Fig. 11.

### 5.9 Electronics

*Required parts: Head Attachment, HOL, CAM (x2), CM (x2), CNC (x1), EM, JUM (x6), MCU (x1), SMD (x3), W3, M2x08 (x8), M2x12 (x2), M3x08 (x3), M3x12 (x7), N_M2 (x10), N_M3 (x10)*

**Fig. 12:**
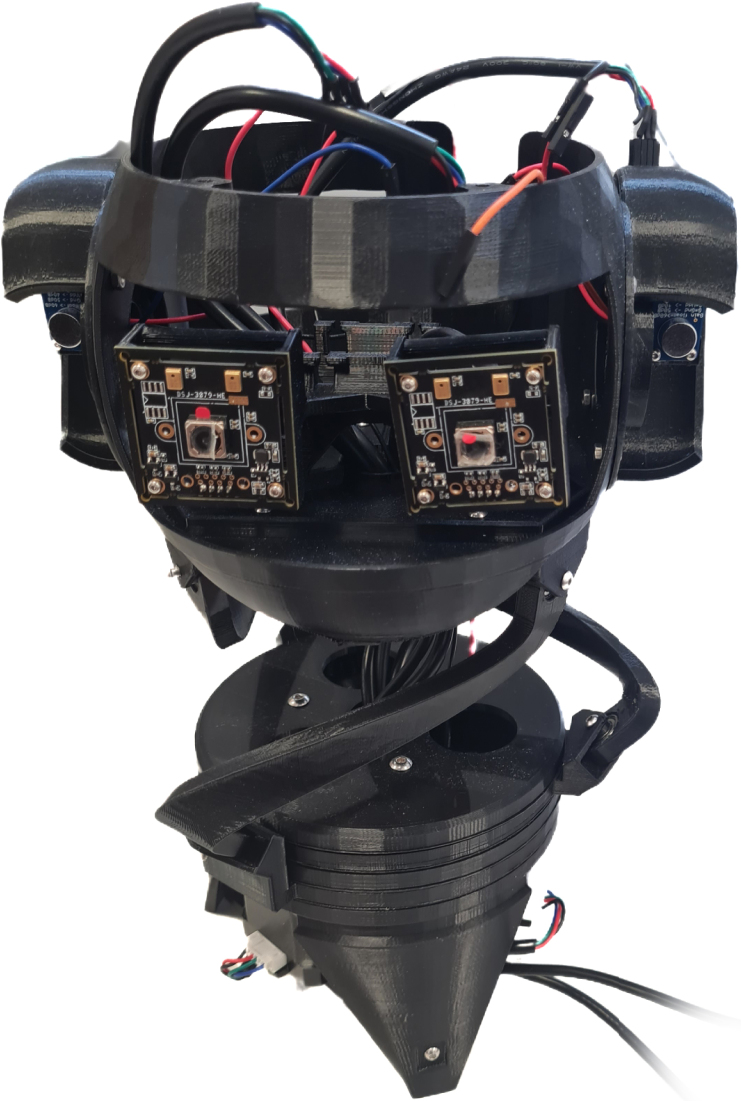
Head assembly with eyes mounted.

**Fig. 13:**
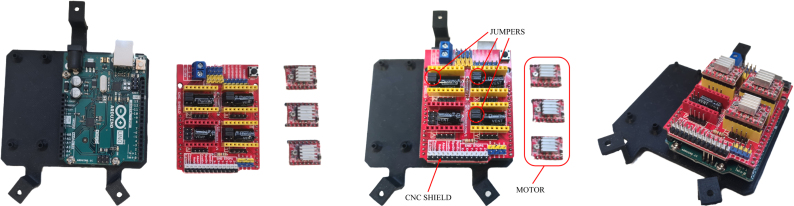
Control electronics (a) Individual parts — Arduino UNO, CNC Shield, 3x motor controllers (b) CNC shield mounted on Arduino with jumpers attached (c) All components assembled.

**Fig. 14:**
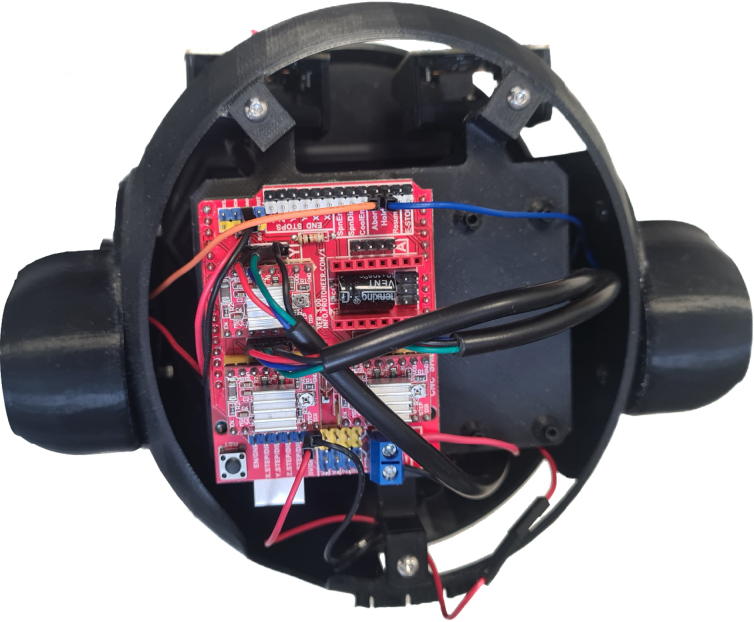
Completed wiring for control electronics.

**Fig. 15:**
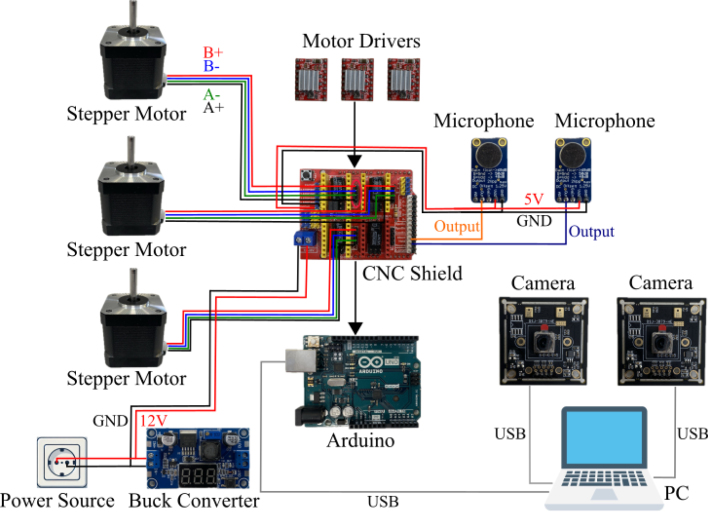
Electrical diagram of BEATRIX.

1. Ensure all M2 nuts are placed in the camera mounts.
2. Place camera mounts onto eye holder so that the square face of the camera mounts point away from the eye holder, and secure them with M2x12 screws.
3. Pass camera cables through the middle of the eye holder and attach them to the cameras.
4. Screw cameras onto camera mounts using M2x08 screws.
5. Mount the eye holder onto head with the M3x12 screws.
6. Attach the Arduino to the HOL with 3x M3x12 screws and M3 nuts.
7. Place CNC on the Arduino.
8. Remove protective film from the base of the heat sinks that came with the motor drivers.
9. Place heat sinks on the HR4988 chip with the fins parallel to the pin headers on the board.
10. Place jumpers on the headers of the Arduino shield to put the X, Y and Z motor channels in 1/8 stepping mode. Further described in [21].
11. Place a motor controller in each of the X, Y and Z positions of the Arduino shield.
12. Use M3x08 screws and nuts to secure HOL to SKL.
13. Connect the 400 mm cables to their respective terminals on the CNC power connector.
14. With the wires going around the back of the eye holder, connect them to the Arduino HAT (positioning the black wire of the motor cables closest to the X, Y, Z labels).
15. Connect the left and right microphone outputs to the “ABORT” and “HOLD” pins of the CNC, respectively.
16. Connect the VDD and GND connections o the microphones to the available 5 V and ground pins of the CNC. The step-by-step process to assembly the electronic components is illustrated in Figs. 11, 12, 13 and 14.
17. Electrical diagram of the overall robotic system is given in Fig. 15

## 6 Operation instructions

The operation of the robot is fairly straightforward with the provided code, available in OSF (https://osf.io/t8egc/) repositories, which includes robot motion functions, MATLAB and Python codes for testing the motors, cameras and microphones.

### 6.1 Motor functions and testing

**Fig. 16:**
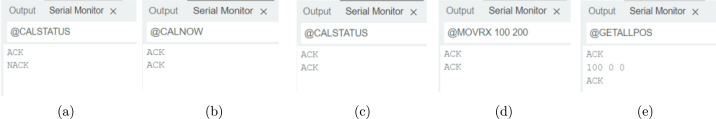
Example outputs from motors commands in the serial terminal of Arduino IDE. (a) Calibration status command before calibrating the robot. ACK: correct command; NACK: robot not calibrated. (b) Calibration command to set the initial/home position. ACK: correct command; ACK: successful calibration. (c) Calibration status command after calibrating the robot. ACK: correct command; ACK: robot is calibrated. (d) Relative movement of motor X by 100 steps and speed of 200. ACK: correct command; ACK: successful motor movement. (e) Get current position of all motors (x =100 y =0 z =0) after the movement command. ACK: correct command; successful command execution.

To actuate the stepper motors located in the robot base, it is crucial to set the correct current limit in each motor driver (A4988 modules in the CNC board). This process ensures optimal performance and avoid damage before testing the motor functions. The process of setting up the current limit requires adjusting the potentiometer located in the A4988 motor driver module to the appropriate value. To complete this configuration process, follow the steps below:

• Power the stepper motors and connect the Arduino Uno microcontroller to your PC using the USB cable.
• Set the current limit of the A4988 motor driver by adjusting the potentiometer. The potentiometer prevents the current flowing through the stepper motor from exceeding its maximum rated current. You can get the current limit by measuring the $V_{ref}$ voltage (V) on the potentiometer. $R_{CS}$ measured in ohms (Ω) is the current sensing resistor with a value of 0.068 Ω. The current limit $I_{max}$ measured in amperes (A), which relates to the motor’s current rating, is determined by the reference voltage as follows:
  (1) $$I_{max}=\frac{V_{ref}}{8\times R_{CS}}\;\text{A}$$
  (2) $$V_{ref}=I_{max}\times 8\times R_{CS}\;\text{V}$$
  To avoid the motors heating up quickly you can reduce the $V_{ref}$ value by dividing by 2.5
  (3) $$V_{ref}=\frac{I_{max}\times 8\times R_{CS}}{2.5}\;\text{V}$$
  The value of $I_{max}$ (current rating) can be found in the corresponding datasheet of the stepper motor. The value of $V_{ref}$ should be between 0.35 V and 0.45 V to drive the motors without heating them up.
• The Arduino Uno microcontroller contains the firmware with the list of functions to allow users to communicate with the robot and control the motors (robot neck). The Arduino code with the full list of functions can be downloaded from the OSF repository (https://osf.io/t8egc/). Table 4 shows an example of the functions and their format used to test the robot motions. When the commands are tested on the *Serial Monitor* of the Arduino IDE, the serial communication needs to be set to *Carriage Return* and the baud rate to 9600. All the motor functions programmed in the Arduino Uno board return ACK/NACK outputs for monitoring purposes. The first output ACK/NACK indicates that the command was received correctly/incorrectly, respectively. The second output ACK/NACK indicates whether the command was performed correctly/incorrectly, respectively. Examples of this output process is shown in Fig. 16.

**Table 4:** Example of functions and their format in the Arduino Uno microcontroller for motor control.

Command	Explanation
@CALSTATUS	Returns the current status of the robot calibration.
@CALNOW	Calibrate the robot setting the home position (x=0, y=0, z=0) to the current robot position. The robot must be calibrated before running any other motor function.
@GETALLPOS	Returns the position values of all the motors.
@MOVHOME	Move all motors to the calibrated/home position.
@ENMOTORS <ON/OFF>	This function is needed to driver the motors. ON: Enable the motors. OFF: Deactivate the motors.
@MOVRX <stepSize><speed>	Relative movement of the motor connected to the X axis on the CNC shield with *stepSize* (starting from the current motor position) and *speed* values. Example: @MOVRX 100 200 Applies 100 steps to the motor in the X axis at 200 speed value.
@MOVAX <stepSize><speed>	Absolute movement of the motor connected to the X axis on the CNC shield with *stepSize* (starting from the home position) and *speed* values. Example: @MOVAX 150 50 Applies 150 steps to the motor in the Y axis at 50 speed value.
@MOVRALL <stepSizeX stepSizeY stepSizeZ><speedX speedY speedZ>	Relative movement of all motors (X, Y, Z) connected to the CNC shield with <stepSize> (starting from the current motor positions) and <speed> values. Example: @MOVRALL 50 100 150 100 50 200 Moves the motors X, Y and Z by 50, 100 and 150 steps at speeds of 100, 50 and 200 speed values, respectively.
@MOVAALL <stepSizeX stepSizeY stepSizeZ><speedX speedY speedZ>	Absolute movement of all motors (X, Y, Z) connected to the CNC shield with <stepSize> (starting from the home position) and <speed> values. Example: @MOVRALL 100 300 150 50 150 100 Moves the motors X, Y and Z by 100, 300 and 150 steps at speeds of 50, 150 and 100 speed values, respectively.

### 6.2 Camera testing

BEATRIX has two cameras that connect via USB, they can be tested by connecting them to a computer or laptop. The cameras should appear as “UC60 Video” under the “Imaging Devices” tab, under Cameras for Windows. In Linux this can be found using the ‘lsusb’ command in the terminal. Once the cameras have had their connections verified, we can then test the video output. This process can be carried out using a basic MATLAB script provided in the OSF repository. The code provided starts a new figure and window to display the current video feed. It can then be closed with either the ‘q’ key or the escape key. The user should delete the current video device in the script and click their connected device from the MATLAB drop down menu. This ensures that the correctly indexed device is connected for the script.

**Fig. 17:**
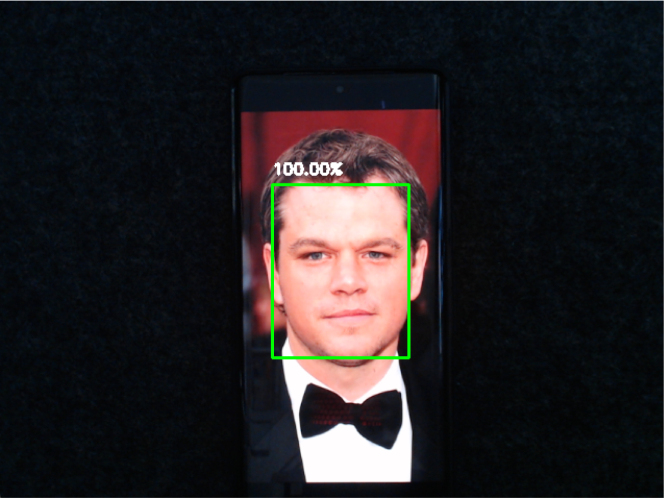
The webcam output of BEATRIX to test either the Haar cascades or MTCNN computer vision algorithms.

Recognition of faces is achieved by using either the built in Haar cascade classifier of the OpenCV package or with the Multi-Task Cascaded Convolutional Neural Network (MTCNN) package [22]. Both packages recognize faces in different ways, but achieve the same goal. Haar cascades use a sequential series of classifiers applied to a region of an image to decide if the region is a face or not. A single-shot approach is often used to discard non-face regions, and then each region within the face has an increasing number of features to compute. This means that the algorithm is computationally efficient, but comes at the cost of being light and pose sensitive. MTCNN is a environmentally robust method that returns a likelihood of the object being a face. This uses a series of 3 CNNs (P-Net, R-Net, O-Net) to recognize the features and pose of the face. This increases accuracy and reliability at the cost of computational efficiency. BEATRIX can be used to teach both forms of computer vision helping students and beginners to learn the foundations. The code for both of these can be tested on a laptop or desktop webcam prior to implementation on BEATRIX. An example of the desktop output is shown in Fig. 17.

**Fig. 18:**
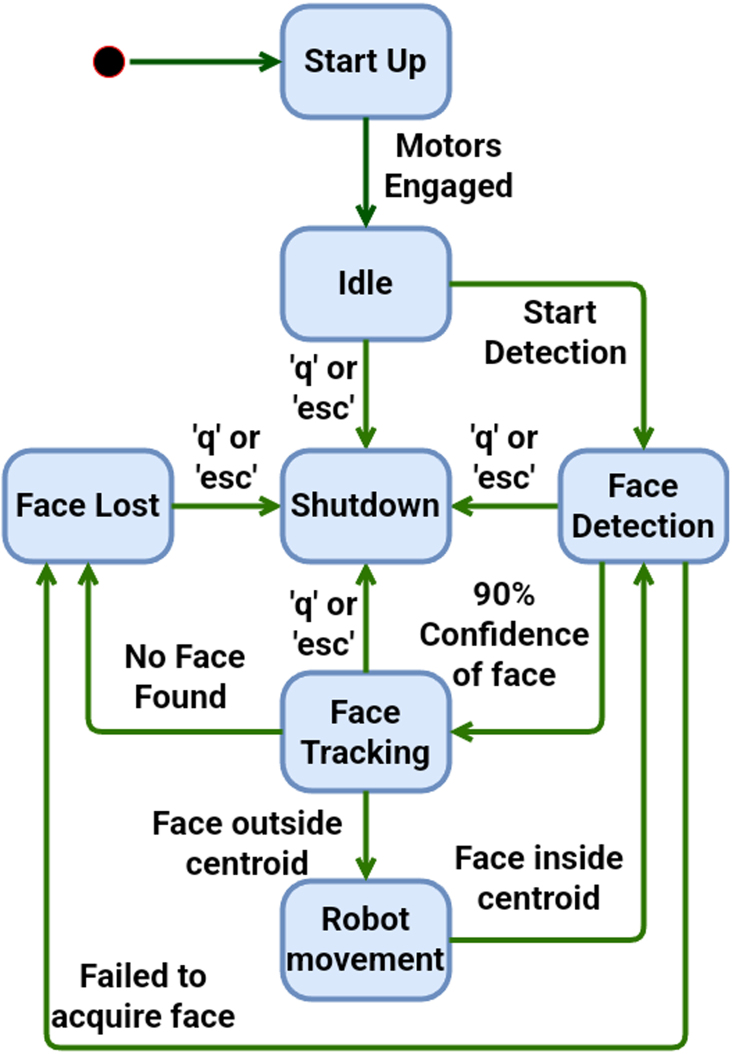
A simple state machine for operating BEATRIX with computer vision for face detection and following.

The motion control of BEATRIX is a state space machine. Position control utilizes the centroid of the image to ensure BEATRIX follows a face. The state machine is shown in Fig. 18. BEATRIX receives relative position changes relative to the position of the face in the frame. The position of the face is provided in terms of X and Y coordinates. This provides an easy transformation for the movement of the motors by using the Z motor for the Y direction and the combination of all three for the X movement.

**Fig. 19:**
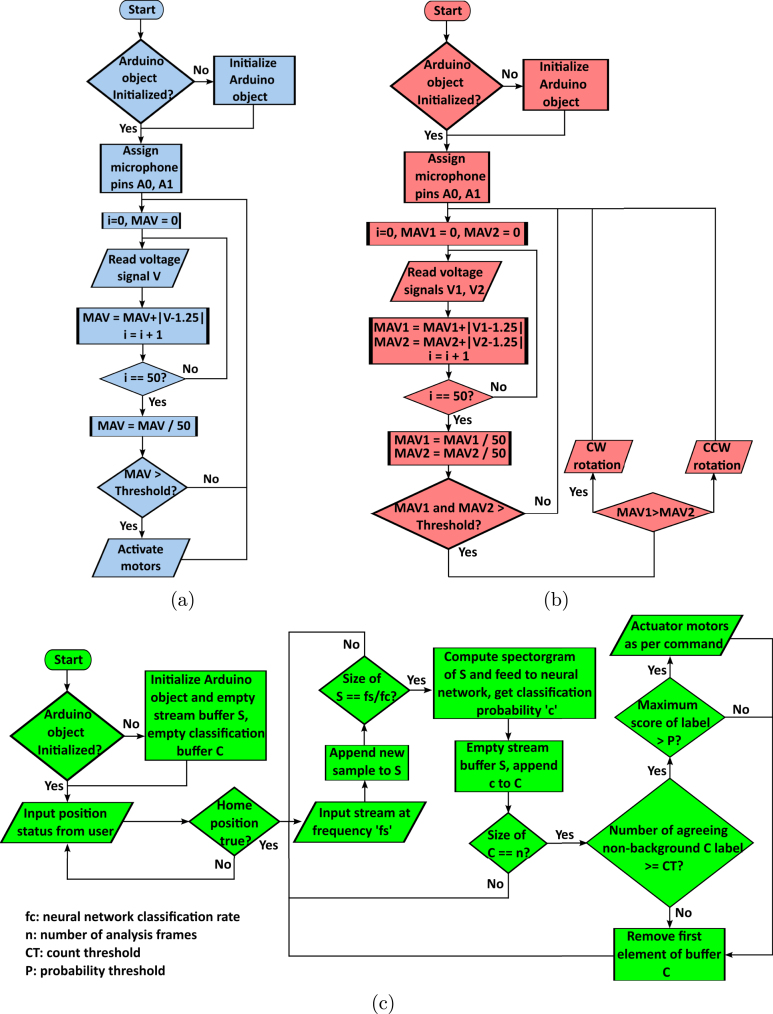
Audio logic flow charts (a) Audio detection (b) Audio tracking (c) Audio recognition.

### 6.3 Microphone testing

The robot features two microphones connected to pins A0 and A1 on the Arduino UNO board. MATLAB’s Arduino hardware plugin can be employed to read voltage signals from these analog pins. An external audio source, such as music from a mobile phone or laptop, demonstrates the changes in the microphones’ outputs. Given the slow refresh rate of the onboard microphones for intricate audio recognition, an external microphone, such as a personal computer microphone, can be utilized to input audio commands into MATLAB, enabling the robot to respond appropriately. Recognition of audio voice commands is achieved using MATLAB’s Audio Toolbox [23] and Deep Learning Toolbox [24]. For simple recognition tasks, such as audio detection and audio tracking, onboard microphones feed input audio as a voltage signal which is later digitized using the board’s ADC. Audio detection computes the mean absolute value (MAV) of the most recent n samples for both microphones, and if the MAV exceeds a certain threshold for any microphone, an audio source is detected. Audio tracking is achieved by computing the differential of the segmented window MAVs of the two onboard microphones. The robot motors are actuated using angular commands whose absolute reference is the robot’s standard position. Positive angular commands lead to clockwise motion, while negative angular commands yield counterclockwise motion. For complex audio recognition tasks, an external microphone with a sufficiently high refresh rate is used instead of the onboard microphones to feed the digitized signal to MATLAB. The input audio stream is segmented into analysis windows, for which the spectrogram is computed and fed to MATLAB’s toolbox pretrained classifier. If the number of a non-background label in the most recent n windows exceeds a count threshold and the maximum probability for that label also exceeds a probability threshold, the motors are actuated in accordance with that label. A **Stop** command disables motors, a **Right** command makes the robot rotate clockwise, and a **Left** command makes the robot rotate counterclockwise. Fig. 19 illustrates the logic flow chart of the audio detection, tracking and recognition tasks.

## 7 Validation and characterization

The BEATRIX robot platform has been adopted as a teaching aid in the Department of Electronic and Electrical Engineering, the University of Bath, Bath, UK. This robot has been used in project weeks, where students need to solve a variety of tasks related to robot control, computer vision and audio processing with easy, intermediate and advanced levels of complexity. This robot has also been used as a platform for research and development for final year projects in the MSc in Robotics and Autonomous Systems, at the University of Bath. These activities allow the students to put into practice their theoretical knowledge, skills, motivate their curiosity and teamwork but also challenging them to solve problems. The BEATRIX robots has also been used as an outreach tool for Science Technology Engineering and Mathematics (STEM) at primary and secondary school levels in the UK. The accessible and open hardware and software nature of BEATRIX enables customization to the firmware and teaching concepts in robotics, humanoid–robot interaction, human–computer interfaces, sensing, computer vision, audio processing, machine learning and electronics. The follow paragraphs show an example of the task descriptions that undergraduate students need to solve in their project weeks using the BEATRIX robot platform.

### 7.1 Motor control with BEATRIX

*Objective:* Develop a program, for example in Python or MATLAB, to control the orientation of the robot head using the functions available in the Arduino microcontroller from Table 4.

*Tasks:*

**Fig. 20:**
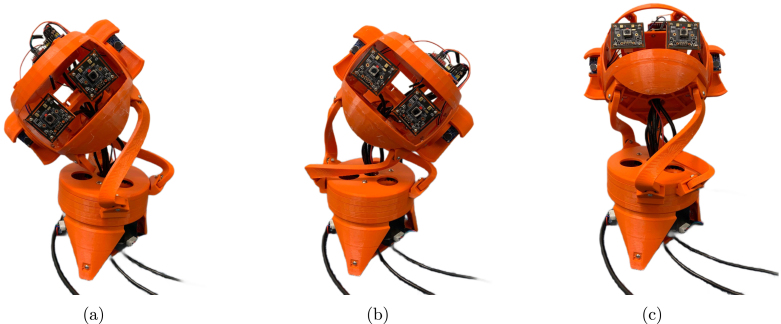
Motion testing: (a) Beatrix turning right with 300 steps for x (counterclockwise) and z (clockwise) motors; (b) Beatrix turning left with 300 steps for x (clockwise) and z (counterclockwise) motors; (c) Beatrix tilting upward with 700 steps for y and 350 steps for z (both counterclockwise).

• Direction control: The direction of motor rotations determines the resulting head movement. Tilting the head to the left requires the x-axis motor to rotate counterclockwise and the z-axis motor to rotate clockwise by the same number of steps, with the reverse occurring for tilting to the right. For upward movement, both the y-axis and z-axis motors rotate counterclockwise. However, to maintain an upright posture, the z-axis motor must rotate approximately half as many steps as the y-axis motor. See Fig. 20, Fig. 20.
• Displacement control: Head displacement is managed by controlling the number of motor steps while maintaining the previously defined step ratios between motors for each pose. The maximum safe number of steps per pose is approximately 300 steps for right/left motion involving the x and z motors, and approximately 700 and 350 steps for the y and z motors, during upward motion. See Fig. 20(c).

**Fig. 21:**
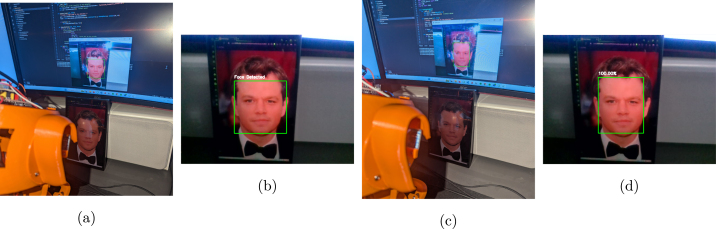
(a) Detection using Haar Cascade. (b) Detection from Haar Cascade. (c) Detection using MTCNN. (d) Detection from MTCNN. The comparison highlights the differences in detection methods between the Haar Cascade and MTCNN for face detection.

**Fig. 22:**
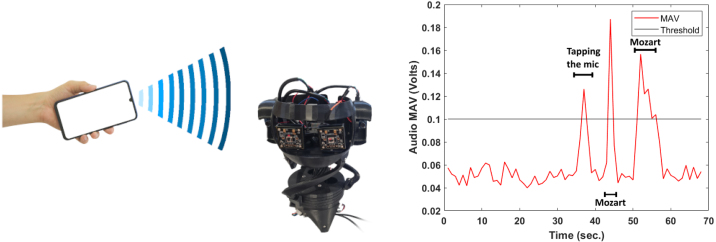
Audio detection trial with outputs visualized, recorded while tapping the microphone once and playing Mozart music twice at different time instants.

### 7.2 Object tracking with BEATRIX

#### 7.2.1 Implementing face detection with Haar cascade classifiers

*Objective:* Use a computer vision method such as Haar cascade classifiers or MTCNN to detect faces in a controlled environment (e.g., consistent lighting, minimal background clutter, frontal faces).

*Tasks:*

• Setup: Install OpenCV and familiarize yourself with its basic functionalities.
• Face detection: Implement face detection using predefined methods in OpenCV such as Haar cascade classifier or MTCNN. You can also explore and implement other methods available in OpenCV. Test the implementation on static images to ensure it can accurately identify faces. The returned image can have a rectangle drawn around it with the detected face. If a CNN is used, the probability can also be returned such as in Fig. 21.
• Robot control: Develop a simple control system where the robot moves in a predetermined direction upon detecting a face. For instance, the robot could move for a few seconds once a face is detected and stop when no face is detected.

### 7.3 Audio detection with BEATRIX

#### 7.3.1 Robot reacting to sound detection

*Objective:* Make the robot react to external loud sounds with a relatively high amplitude compared to the background noise. The robot should dance by rotating one of the motors left and right starting from a known home position without exceeding the allowable rotation threshold of the mechanical frame.

*Tasks:*

• Adjusting the Robot’s Home Position: The MATLAB code must first prompt the user to adjust the robot’s pose to match the home position, the user then should answer MATLAB’s inquiry by typing true and pressing ENTER.
• Audio Analog Read: The MATLAB code must then start a loop in which it received fresh analog inputs from the microphone.
• Signal Processing: The MATLAB code will group a number of successive samples to form processing windows. The Mean Absolute Value (MAV) of each window should be computed after removing the background noise offset.
• Robot control (dancing action): If the MAV of a window exceeds a specific threshold, the robot should start to dance by rotating one of the motors left and right by a specific number of steps starting from the home position using MATLAB’s playtone() function. Students are expected to adjust the frequency and duration of the tone accordingly. The output from a sample test run is illustrated in Fig. 22.

## Ethics statements

The authors confirm that this work did not involve human subjects.

## CRediT authorship contribution statement

**Gorkem Anil Al:** Writing – original draft, Visualization, Validation, Software, Project administration, Methodology, Investigation. **Nicholas Hedworth:** Validation, Software, Methodology, Investigation. **Douglas Tilley:** Writing – original draft, Visualization, Validation, Software, Project administration, Methodology, Investigation. **Samer Ahmed:** Writing – original draft, Visualization, Validation, Software, Methodology, Investigation. **Richmond Afeawo:** Writing – original draft, Visualization, Validation, Software, Methodology, Investigation. **Uriel Martinez-Hernandez:** Writing – review & editing, Writing – original draft, Visualization, Validation, Supervision, Software, Resources, Project administration, Methodology, Investigation, Formal analysis, Conceptualization.

## Declaration of competing interest

The authors declare that they have no known competing financial interests or personal relationships that could have appeared to influence the work reported in this paper.
